# Prevalence and Risk Factors of Low Health Literacy: A Community-Based Study in Shanghai, China

**DOI:** 10.3390/ijerph14060628

**Published:** 2017-06-12

**Authors:** Ying Wu, Lu Wang, Zhongyuan Cai, Luqi Bao, Pu Ai, Zisheng Ai

**Affiliations:** 1 Postdoctoral Research Station of Medicine, School of Medicine, Tongji University, Shanghai 200092, China; wuying19890321@gmail.com; 2 Baoshan Center for Disease Control and Prevention of Shanghai, Baoshan 201900, China; sd85588210@126.com (L.W.); cyzzjq@163.com (Z.C.); a412926008@163.com (L.B.); 3 Department of Clinic Medicine, Anhui Medical University, Hefei 230022, China; aipu1995@126.com; 4 Department of Medical Statistics, School of Medicine, Tongji University, 1239 Siping Road, Yangpu District, Shanghai 200092, China

**Keywords:** health literacy, prevalence, health education, knowledge, China

## Abstract

*Background:* Health literacy is an increasingly important public health concern. However, little is known about the health literacy of general public in China. The aim of this study was to evaluate the prevalence of low health literacy and demographic associations in Shanghai, China. *Methods:* This study was a community-based cross-sectional health survey utilizing a multi-stage random sampling design. The sample consisted of 1360 individuals aged 15–69 years with the total community-dwelling Chinese as the sample frame. Health literacy was measured by a questionnaire developed on the basis of a national health literacy manual released by the Chinese Ministry of Health. Multiple logistic regression models were used to identify whether common socio-demographic features were associated with health literacy level. *Results:* The prevalence of low health literacy was 84.49% (95% CI, 82.56% to 86.41%). The prevalence of low health literacy was negatively associated with the level of education, occupation, and annual household income, but was not associated with gender, age, or the presence of non-communicable chronic disease. *Conclusions:* Simplifying health services, enhancing health education, and promoting interventions to improve health literacy in high-risk populations should be considered as part of the strategies in the making of health policy in China.

## 1. Introduction

Health literacy has been defined by the US Institute of Medicine (IoM) as “the degree to which individuals have the capacity to obtain, process, and understand basic health information and services needed to make appropriate health-related decisions and follow instructions for treatment” [1,2]. This definition was further enhanced by WHO to “the cognitive and social skills which determine the motivation and ability of individuals to gain access to, understand and use information in ways which promote and maintain good health” [3]. Patients with adequate health literacy can read, understand, and act on health care information. Numerous studies indicated that low health literacy was associated with worse health outcomes and higher rates of hospitalization [4,5,6,7,8]. Health care providers routinely expect health care consumers to have adequate health literacy. There is disparity, however, between the health literacy required to maintain good health and the health literacy level of many patients. For example, a study conducted at two urban public hospitals in America [9] examined the functional health literacy level of patients with hypertension or diabetes. Only 39% of 402 patients with hypertension and 45% of 114 patients with diabetes had health literacy skills adequate to function in the health care settings. A systematic review of the prevalence of limited health literacy in the United States found that the weighted prevalence of marginal health literacy was 20% and of low health literacy was 26% [10]. A national survey in England of the literacy skill found that 16% of working age adults had limited literacy [11]. 

Although it has been documented that inadequate health literacy is common in developed countries, the health literacy level of Chinese people has not been well described. Compared with the long and comprehensive history of health literacy studies in a wide variety of settings in the United States, only a limited number of health literacy studies have been carried out in China. In 2008, the first national survey of the public health literacy status was conducted by the Chinese Ministry of Health in the mainland of China, profiled the comprehensive health literacy of 79,542 Chinese aged 15–69 years and found that only 6.48% of Chinese residents have adequate health literacy [12]. The health literacy was assessed using a questionnaire based on an announcement in which 66 items of basic health-related knowledge and skills were formally defined by the Chinese Ministry of Health [13,14]. However, it has been suggested that, in addition to health-related knowledge, poor ability to gain access to and understand health information and services is common in developing countries [15]. Hence, in 2012, the Chinese Health Education Authority issued a new strategic plan to assess health literacy nationally, which contained expanded components related to ability to gain access to and use health information to accomplish specific health literacy tasks [16]. Therefore, our study focused on Chinese residents’ health literacy assessed by this instrument whose reliability and validity had been well established [17]. In addition, China is a large country with a population of about 1.399 billion [18], so the residents’ health literacy status may be associated with geographic areas due to unbalanced economic development. Compared with the national prevalence of a low health literacy of 93.52%, a study conducted in the Jiangsu province of China in 2010 documented that the prevalence of low health literacy in that area was 47.5% [19], and a study conducted in Beijing, China, evaluating the communicable diseases health literacy of Beijing residents showed that the percentage of residents with inadequate communicable diseases health literacy was 59% [20]. To eliminate the geographic disparity, surveillant programs, efficient interventions, and appropriate education strategies should be developed for different areas. Most of the studies attempting to assess health literacy and investigate the association between health literacy and potential risk factors in the general public were conducted in Hong Kong or Taiwan, only a few have been conducted in the mainland of China, and none of them was in Shanghai, China. The objectives of this study were to (1) examine the health literacy in the residents of Baoshan District of Shanghai, China, in 2016 and (2) examine the socio-demographic factors associated with health literacy among this population.

## 2. Materials and Methods

### 2.1. Study Population and Sampling Design

**Figure 1:**
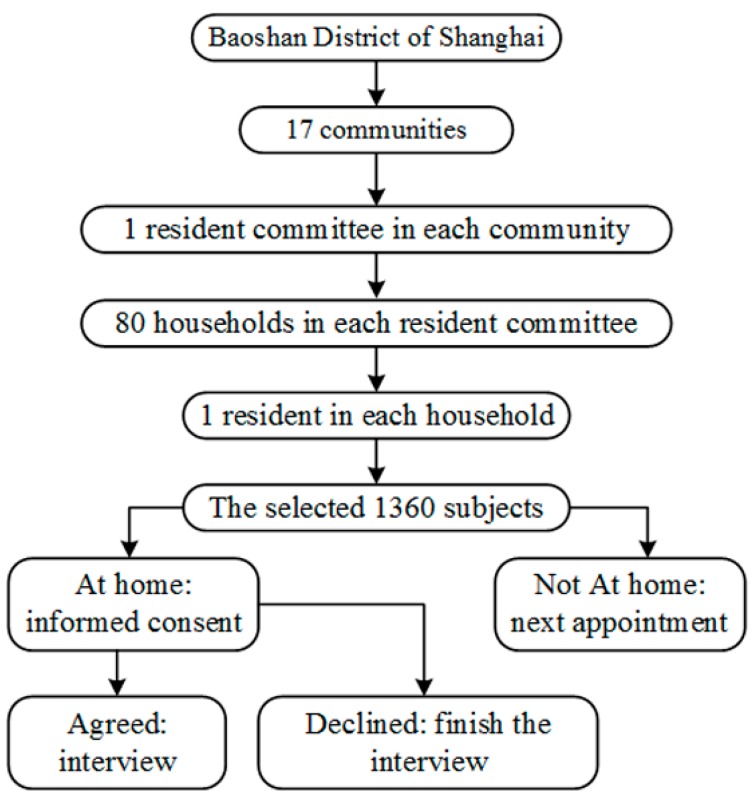
Flow chart for participants sampling and interview in the study on health literacy in a 15–69-year-old population, Shanghai, China, 2016.

This study was conducted in Baoshan District of Shanghai, which had a total population of 1.62 million [21]. The study population were residents aged 15–69 years and had been living in Baoshan District of Shanghai for more than 1 year. We used a community-based cross-sectional multi-stage random sampling frame, as shown in Figure 1. The population in Baoshan District of Shanghai was divided into 17 communities and one resident committee in each community was chosen at random. The sample size of each chosen resident committee was calculated by the formula

$$N=\frac{Z_{1-\alpha /2}^{2}\times \pi (1-\pi )}{\delta^{2}}\times deff$$

where α was the significance level, $Z_{1-\alpha /2}$ was the (1−α/2)-quantile of the standard normal distribution, π was the percentage of people with low health literacy, δ was the maximum permissible error, and deff was the design effect of complex sampling used to adjust the effectiveness loss due to complex sampling instead of simple random sampling. Since the national health literacy survey in 2008 showed that the prevalence of low health literacy among city residents was 89% [12], the prevalence in this study was expected to be π=0.89, maximum permissible error δ=0.1π, significance level α=0.05, $Z_{1-\alpha /2}=1.96$, and the design effect of complex sampling was deff=1.5. Then, the required sample size of each resident committee was N=71.22. Taking into consideration a non-response rate of 10%, the actual sample size in each subgroup increased to 71.22/0.9=79.13, rounded to 80. Then, 80 individuals were chosen as participants at random from each resident committee. That is, 80 households were randomly selected from each resident committee, and one member from each chosen house whose day of birth was closest to the 15th was selected as the respondent. If the selected member refused to complete the questionnaire, unselected members were not allowed to complete it as a substitution. Finally, the total sample size of 17 resident committees was expected to be 80×17=1360. Participants were excluded from the survey if they were military personnel, prisoners, or unable to communicate in Mandarin.

### 2.2. Data Collection

The survey was conducted from January to April in 2016. Participants were interviewed face-to-face to complete a questionnaire developed with reference to a national health literacy manual entitled “Basic Knowledge and Skills of People’s Health Literacy” released by the Chinese Ministry of Health [16]. The questionnaire was divided into two parts: the first part was designed to collect socio-demographic characteristics (gender, age, education level, occupation, and average household income) and the presence of non-communicable chronic disease (any one of 11 non-communicable chronic diseases including lung disease, liver diseases, renal disease, peptic ulcer disease, chronic back-pain, arthritis, diabetes, neurological disorder including stroke, allergy, depression, and cancer); and the second was the health literacy scale. A total of 50 questions in the health literacy scale were categorized into three subscales representing (1) basic knowledge and concepts; (2) lifestyle; and (3) health-related skills covering aspects including scientific views of health, prevention of acute infectious diseases, prevention of non-communicable chronic diseases, safety and first aid, basic medical care, and health information. The study protocol was approved by the Research Ethic Committee of Shanghai Center for Disease Prevention and Control, and informed consent was obtained from all participants before taking part.

There were four types of questions in the scale: true-or-false questions, single-answer questions, multiple-answer questions, and situation questions. Note that the different types of questions did not represent different dimensions of health literacy. Values were assigned to each of the questions. For true-or-false and single-answer questions, 1 point was assigned for a correct answer, and 0 point were assigned for an incorrect answer. For multiple-answer questions, 2 points were assigned if the response contained all the correct answers and no incorrect ones, and 0 point were assigned otherwise. For situation questions, participants had to read passages and answer single- or multiple-answer questions about it. Every incorrect answer as well as every unanswered question was scored with 0 point. As a sum of scores on all the three subscales, the overall health literacy score ranged from 0 to 65. Participants receiving a score of 52 (which was 80% of the full marks of 65) or less were categorized as having a low literacy level.

Before the survey was performed, six survey teams each comprising a coordinator, four investigators, and a quality controller were established. All team members were given training about the sampling method and test instruments. Quality control was given to all team members, as well as a simulated survey assessing their eligibility.

### 2.3. Statistical Analysis

Descriptive analyses were performed for socio-demographic characteristics, the presence of non-communicable chronic disease, and health literacy of all participants. Health literacy by socio-demographic characteristics and presence of chronic disease was also described. Comparisons of health literacy scores among subgroups by participant characteristics were conducted using one-way analysis of variance. Comparisons of prevalence of low health literacy among subgroups were conducted using chi-square tests. Stepwise logistic regression was conducted to identify whether socio-demographic and health variables were associated with health literacy level. During the variable selection process, a significance level of 0.05 is required to allow a variable into the model, and a significance level of 0.10 is required for a variable to stay in the model. All analyses were conducted with SAS software, version 9.4 (SAS Institute, Cary, NC, USA). Multiple imputations were used for imputing missing values for the independent variables. A significance level of 0.05 was used for hypothesis tests.

## 3. Results

**Figure 2:**
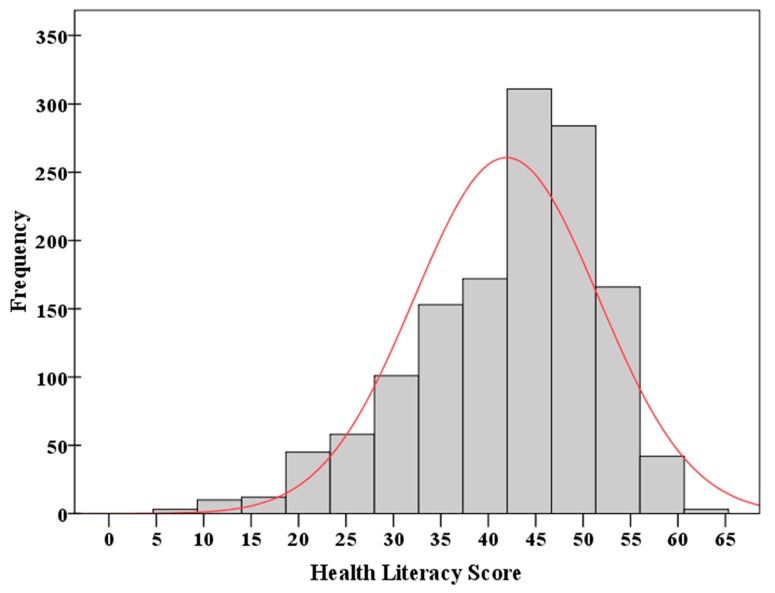
Histogram of the health literacy scores obtained from 1360 participants. The curve represents a normal curve with the mean and standard deviation estimated from the sample.

**Figure 3:**
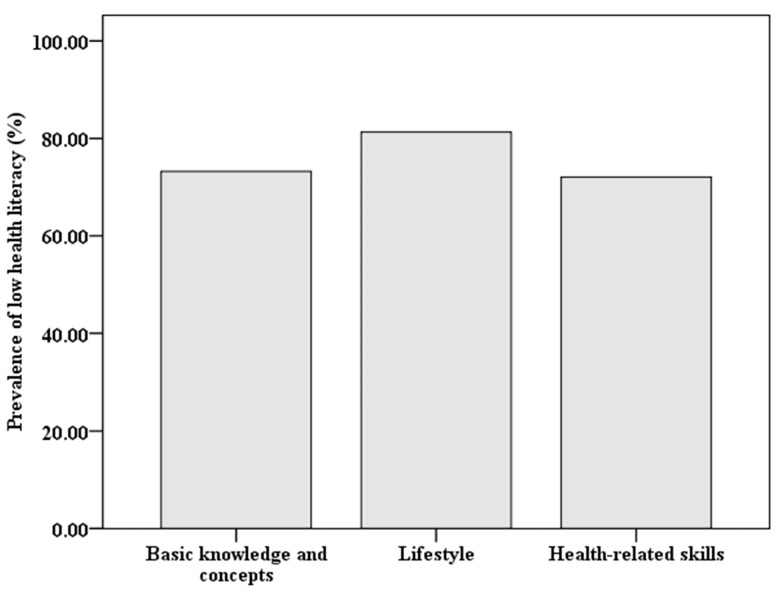
Prevalence of low health literacy measured by three subscales: basic knowledge and concepts, lifestyle, and health-related skills.

A histogram of the health literacy scores (ranging from 0 to 65) obtained from 1360 participants is given in Figure 2. The distribution of scores was slightly left-skewed and being non-normal. The percentages of participants who scored lower than 40%, 60%, and 80% of the full score 65 were 7.21%, 31.40%, and 84.49%, respectively. In terms of the study, the prevalence of low health literacy was 84.49% (95% CI, 82.56% to 86.41%). The prevalence of low health literacy measured separately by three subscales is shown in Figure 3.

**Figure 4:**
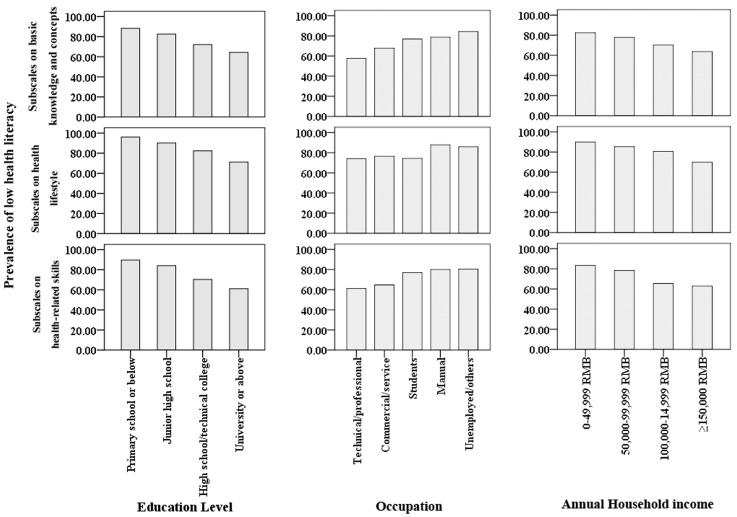
Prevalence of low health literacy by three subscales and characteristics.

Since multiple risk factors may be involved, several risk factors must be controlled for simultaneously analyzing characteristics associated with low health literacy. A multiple logistic regression model was considered in this study. The results of stepwise logistic regression analyses are presented in Table 1. After adjusting for potential confounders, the level of health literacy was significantly associated with occupation, education level, and annual household income. After controlling for all other risk factors in the logistic regression model, residents with an education level of high school/technical college (OR = 0.190, 95% CI = 0.045–0.806, *p* = 0.024) and university or above (OR = 0.140, 95% CI = 0.033–0.599, *p* = 0.008) were 81% and 86%, respectively, less likely to have a low health literacy than residents with an education level of primary school or below. Manual workers (OR = 2.413, 95% CI = 1.302–4.470, *p* = 0.005) and the unemployed/others (OR = 2.470, 95% CI = 1.237–4.934, *p* = 0.010) were 141% and 147%, respectively, more likely to have low health literacy than residents whose occupation was technical or professional. Residents with an annual household income equal to or higher than 150,000 RMB (OR = 0.432, 95% CI = 0.213–0.874, *p* = 0.020) were 67% less likely to have low health literacy than residents with an annual household income lower than 50,000 RMB. The prevalence of low health literacy measured by subscales in subgroups categorized by the three risk factors is presented in Figure 4.

## 4. Discussion

In our study, conducted in Shanghai, China, 84.49% of the residents had low health literacy, slightly lower than the national average of 93.52% reported by the Chinese government in 2008 [12]. The decrease in prevalence of low health literacy might be due to rapid economic development, resulting in enhanced education, as well as improved healthcare service. However, this figure was much higher than the figure 26% reported in a systematic review of 31,129 participants in the United States [10]. A prevalence of low health literacy in general public of 47.5% was reported in a study conducted in the Jiangsu province of China in 2010, in which the low health literacy was defined as score less than 60% of the total score [19]. By resetting the low health literacy in our study to score less than 60%, a prevalence of low health literacy of 31.5% was obtained, lower than that of the Jiangsu province of China. This suggested that low health literacy was still prevalent in China, and the prevalence was likely to be even higher in many other regions within the mainland of China than in Shanghai. The high prevalence presented a challenge to researchers, community organizations, health care providers, and policy-makers interested in improving the health of Chinese by improving their health literacy.

Results from our study showed that the prevalence was particularly high amongst residents: (1) who only completed education of junior high school and below; (2) whose annual household income was 49,999 Yuan and below; (3) who were mainly engaged in manual labor; and (4) aged 60 years and above. After adjusting for all other potential confounders in the logistic regression model, education level, occupation, and annual household income were factors associated with a prevalence of low health literacy. Our findings were consistent with several large-scale surveys of adult health literacy, which reported that the most common socio-demographic features associated with health literacy were education level, age, ethnicity, geographic location, and income [8,22,23,24,25,26,27,28]. Our study demonstrated that, after adjusting for potential confounders, the prevalence of low health literacy was significantly associated with occupation, education level, and annual household income. Healthcare providers serving communities with high risk of low health literacy may then target and implement additional supports and strategies to improve health literacy. For example, increasing residents’ access to health information, offering patient counseling with health educators, and using plain language to ensure understanding of health information. A suggestion is that appropriate interventions to improve public health literacy level should pay attention to residents who are mainly engaged in manual labor. From practical viewpoints, it is easier to identify residents by occupation than by education level or income. In addition, clinicians might be able to identify patients with low health literacy by these characteristics, and then overcome communication barriers to ensure that such patients receive high-quality health care. Such changes have the potential to improve the public health literacy and address the health and health literacy disparities that exist today.

Somewhat surprising was the lack of correlation between age and health literacy in the multiple logistic regression model, while a significant association was shown in the bivariate analysis (Table 2). However, the result of the chi-square test examining the relationship between age and education level suggested that a resident’s age was correlated with their education levels (*χ*^2^ = 374.86, *p* < 0.001). About 63.1% of the residents aged 60 years or older had only completed education of junior high school or below, whereas only 8.2% of residents aged 15–35 years had education levels of junior high school or below. Therefore, the high prevalence of low health literacy among the elderly may be due to their low level of education.

It was noteworthy that the prevalence ratio of residents with education level of High school/technical college to the general residents was 84.40%/84.49% = 99.89%. In other words, high education completion in this district of Shanghai only decreased the prevalence of low health literacy by 0.11% in contrast to that of 58.85% in the United States [10]. Results from the subscale for health-related skills suggested that Chinese residents commonly did not have an adequate capacity to understand health information and services, and use them to perform basic health-related tasks, even if some education and services had been given. Therefore, it is essential to enhance health education and services at a level that does not exceed the understanding ability of general residents.

In addition to the comprehensive health literacy, three elements of health literacy were also separately assessed in this study. From Figure 3, we found that the prevalence of low health literacy related to health lifestyle was comparatively higher than the other two components of comprehensive health literacy: basic health knowledge and concepts and health-related skills. This indicates that Chinese people may lack awareness of the concept that a healthy lifestyle is helpful for maintaining health and preventing diseases or injuries rather than just curing them or alleviating their symptoms. Given the fact that China is a developing country, this may be related to the socio-cultural and socio-economic limitations.

Several limitations to this study are worth noting. First, this study presented an estimate of the prevalence of low health literacy in Baoshan District of Shanghai, China. However, the representativeness of this district for the whole city cannot be guaranteed. There may be disparities in socio-demographic or socio-economics as well as other potential factors between this district and the whole city of Shanghai. In addition, the comparability of the results of this study with other countries might not been well ensured due to the homegrown measures of health literacy. Second, although potential risk factors in our model were selected on the basis of existing literature, there might be other unmeasured characteristics that contributed to health literacy not included in the model. Third, according to our multi-stage sampling design, 80 residents were randomly selected from each of the 17 communities in the Baoshan District. The distributions of the residents’ characteristics in the whole sample would be approximately consistent with those in the district population under a reasonable assumption that all of the 17 communities were of equal size. Nonetheless, this assumption might be slightly violated in practice, which might introduce potential bias to the estimated prevalence of low health literacy in the population. Fourth, the demographic variation as well as the association between health literacy and ethnicity could not be well investigated in this study. According to the Shanghai Ethnic and Religions Affairs Committee, the minority nationalities account for about 0.63% of the population in Shanghai, and there are no different ethnic enclaves in this city [29]. Therefore, we failed to obtain sufficient data about the health literacy of the minorities in Shanghai.

## 5. Conclusions

This study provided the prevalence of low health literacy in Shanghai, China, in 2016, using a revised instrument whose reliability and validity has been established. The prevalence was lower than the national average. Mostly in line with previous studies, the prevalence of low health literacy was negatively associated with the level of education, occupation, and annual household income. The findings of this study supported enhancing the health literacy and offered useful implications for the making of health policy in China.

## Figures and Tables

**Table 1:** Odds ratios in favor of low health literacy and 95% CI in the stepwise logistic regression model.

Risk Factor		OR (95% CI)	*p* Value
Education level			
	**Primary school or below (reference)**		
	Junior high school	0.560 (0.127, 2.461)	0.443
	High school/technical college	0.190 (0.045, 0.806)	0.024
	University or above	0.140 (0.033, 0.599)	0.008
Occupation			
	**Technical/professional (reference)**		
	Commercial/service	1.642 (0.995, 2.710)	0.052
	Students	2.276 (0.834, 6.211)	0.108
	Manual	2.413 (1.302, 4.470)	0.005
	Unemployed/others	2.470 (1.237, 4.934)	0.010
Annual household income			
	**¥0–¥49,999 (reference)**		
	¥50,000–¥99,999	0.681 (0.342, 1.355)	0.273
	¥100,000–¥14,999	0.507 (0.255, 1.010)	0.053
	≥¥150,000	0.432 (0.213, 0.874)	0.020

**Table 2:** Health literacy score and the prevalence of low health literacy by participant characteristics.

Characteristics		Number (%) ^a^	Health Literacy Score		Prevalence of Low Health Literacy	
			Mean ± SD	*p* Value	Number (%) ^b^	*p* Value
Gender	Male	648 (47.6%)	41.87 ± 9.75	0.876	547 (84.4%)	0.944
	Female	712 (52.4%)	41.96 ± 9.68		602 (84.6%)	
Age group	15–35	353 (26.0%)	44.07 ± 9.00	<0.001	270 (76.5%)	<0.001
	36–59	662 (48.7%)	42.34 ± 9.06		566 (85.5%)	
	60–69	345 (25.4%)	38.91 ± 10.85		313 (90.7%)	
Education level	Primary school or below	77 (5.7%)	35.39 ± 10.72	<0.001	75 (97.4%)	<0.001
	Junior high school	396 (29.1%)	38.70 ± 9.66		376 (94.9%)	
	High school/technical college	391 (28.8%)	42.32 ± 9.16		330 (84.4%)	
	University or above	496 (36.5%)	45.19 ± 8.66		368 (74.2%)	
Occupation ^c^	Technical/professional	85 (6.2%)	45.57 ± 8.40	<0.001	56 (65.9%)	<0.001
	Commercial/service	598 (44.0%)	43.61 ± 9.64		477 (79.8%)	
	Students	39 (2.9%)	44.36 ± 7.30		33 (84.6%)	
	Manual	460 (33.8%)	40.01 ± 9.38		423 (92.0%)	
	Unemployed/others	178 (13.1%)	38.92 ± 10.09		160 (89.9%)	
Annual household income ^d^	¥0–¥49,999	187 (13.8%)	38.30 ± 9.92	<0.001	176 (94.1%)	<0.001
	¥50,000–¥99,999	491 (36.1%)	40.57 ± 9.51		436 (88.8%)	
	¥100,000–¥14,999	396 (29.1%)	43.18 ± 9.54		322 (81.3%)	
	≥¥150,000	286 (21.0%)	44.87 ± 9.01		215 (75.2%)	
Prevalence of a non-communicable chronic disease						
	Yes	383 (28.2%)	40.32 ± 10.00	<0.001	340 (88.8%)	0.006
	No	977 (71.8)	42.55 ± 9.53		809 (82.8%)	

^a^ percentage in all respondents; ^b^ percentage in subgroups of respondents by characteristics; ^c^ occupations were categorized into 5 groups: technical or professional occupations (i.e., civil servants, teachers and health care workers); commercial or service occupations (i.e., other public institutions’ staff and employees of enterprise); students; manual occupations (i.e., peasants and workers); and unemployed and all other cases (e.g., the unemployed or retired). ^d^ ¥: RMB, Chinese Yuan.
